# Two-Way Minimization: A Novel Treatment Allocation Method for Small Trials

**DOI:** 10.1371/journal.pone.0028604

**Published:** 2011-12-07

**Authors:** Lan-Hsin Chen, Wen-Chung Lee

**Affiliations:** 1 Institute of Epidemiology and Preventive Medicine, College of Public Health, National Taiwan University, Taipei, Taiwan; 2 Research Center for Genes, Environment and Human Health, College of Public Health, National Taiwan University, Taipei, Taiwan; Genentech Inc., United States of America

## Abstract

Randomization is a hallmark of clinical trials. If a trial entails very few subjects and has many prognostic factors (or many factor levels) to be balanced, minimization is a more efficient method to achieve balance than a simple randomization. We propose a novel minimization method, the ‘two-way minimization’. The method separately calculates the ‘imbalance in the total numbers of subjects’ and the ‘imbalance in the distributions of prognostic factors’. And then to allocate a subject, it chooses—by probability—to minimize either one of these two aspects of imbalances. As such, it is a method that is both treatment-adaptive and covariate-adaptive. We perform Monte-Carlo simulations to examine its statistical properties. The two-way minimization (with proper regression adjustment of the force-balanced prognostic factors) has the correct type I error rates. It also produces point estimates that are unbiased and variance estimates that are accurate. When there are important prognostic factors to be balanced in the study, the method achieves the highest power and the smallest variance among randomization methods that are resistant to selection bias. The allocation can be done in real time and the subsequent data analysis is straightforward. The two-way minimization is recommended to balance prognostic factors in small trials.

## Introduction

Random allocation of subjects is a hallmark of clinical trials. The simplest allocation method is the ‘simple randomization’ (complete randomization with equal allocation) where the recruited subjects are assigned to treatment group or control group based entirely on probabilities (say, using random numbers, or computer-generated random variates) [1]. In a large trial, a straightforward simple randomization often suffices to achieve satisfactory balance between the treatment and the control groups—with respect to the total numbers of subjects and to the distributions of prognostic factors [2].

If a trial entails very few subjects and has many prognostic factors (or many factor levels) to be balanced, one may need to resort to a more sophisticated method of ‘minimization’ [3], [4]. The method achieves balance not by probability but by design. Thus it is a more efficient method statistically as compared with the simple randomization [5], [6], [7]. Minimization has one notable drawback, however—its allocation of subjects becomes predictable to some extent. As such, selection bias may arise and the credibility of the trial can be questioned.

In this paper, we propose a novel minimization method, the ‘two-way minimization’. The method separately calculates the ‘imbalance in the total numbers of subjects’ and the ‘imbalance in the distributions of prognostic factors’. And then to allocate a subject, it chooses—by probability—to minimize either one of these two aspects of imbalances. We perform Monte-Carlo simulations to compare the performances of the two-way minimization with five existing randomization methods.

## Methods

### Imbalance Measures

Consider an arbitrary point during the trial. Let 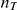 and 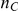 denote the total numbers of subjects allocated to the treatment group and the control group, respectively. The imbalance in the total numbers of subjects is simply 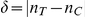.

Suppose that a total of 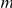 prognostic factors (indexed by 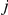) are to be balanced, with a total of 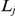 levels (indexed by 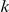) for the 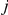 th prognostic factor. Let 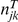 denote the number of subjects allocated to the treatment group, whose 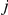 th prognostic factor is at the 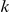 th level. Let 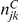 denote the corresponding number of subjects allocated to the control group. We then calculate the proportions (distributions): 
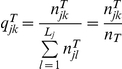
 and 
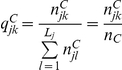
. The imbalance in the distributions of the 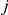 th prognostic factor is defined as 
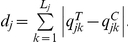
 The overall imbalance in the distributions is a weighted sum of 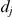
's, that is, 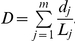


### Two-Way Minimization

At the beginning, we let the trial adopt a simple randomization scheme for allocating subjects. After 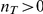 and 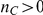, we then shift to two-way minimization.

The proposed two-way minimization is an adaptive randomization procedure [7]. In fact, it is adaptive in two ways: (A1) minimizing the imbalance in the total numbers of subjects (treatment-adaptive), and (A2) minimizing the imbalance in the distributions of prognostic factors (covariate-adaptive). That is,

(A1) minimizing 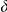:

If 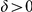, the new subject is to be allocated to the group with fewer subjects already in that group, otherwise, to the treatment and control groups with equal probability.

(A2) minimizing 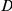:

Let 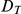 be the overall imbalance in the distributions of prognostic factors if the new subject is allocated to the treatment group, and 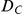, the overall imbalance if allocated to the control group. We then actually allocate the new subject to the treatment group if 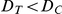, to the control group if 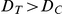, and to the treatment and control groups with equal probability if 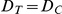.

We let chance dictate which rule (A1 or A2) to use for allocating a new subject. To be precise, we define a parameter 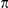 (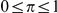). Then, the new subject is allocated according to A1 rule with probability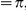 and to A2 rule with probability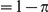. This allocation scheme is equivalent to a scheme that minimizes a weighted sum of 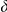 and 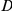, that is, to minimize 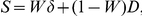 where 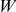
(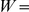
1 or 0) is Bernoulli distributed with parameter 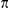. Note that 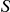 above adopts a ‘stochastic’ weight (the weighting changes each time we allocate a new subject) rather than the usual ‘deterministic’ weight (the weighting is a fixed value). This makes the method robust to any monotone transformation of 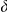 and 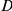. In other words, all allocation schemes that minimize 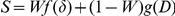 are equivalent for any monotonically increasing 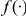 and 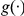, and therefore one need not worry about the functional forms.

Furthermore, the parameter 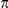 itself need not be fixed throughout the course of allocation, either. It can be made to be responsive to 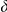, such that when 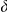 is larger (greater imbalance in the total numbers of subjects), 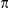 is also larger (higher probability to take action to counter that imbalance). We propose to base 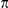 on a simple geometric accrual function: 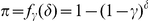, where 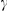
(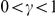) is a tuning parameter. This function has the following properties: (1)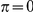 when 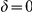; (2)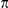 increases as 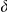 increases; and (3)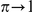 as 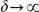. The role of the tuning parameter 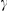 is to govern the accrual rate (an increase in 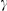 implies an increase in the accrual rate). In the simulation studies that follow, we found that a tuning value of 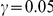 is a satisfactory choice.

## Results

### Simulation Setups

We assume that there are a total of 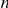 subjects (indexed by 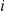) to be allocated and a total of 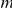 prognostic factors (indexed by 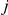) to be balanced with a total of 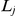 levels (indexed by 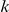, with 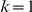 indicating the reference level) for the 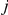 th prognostic factor. Let 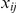 denote the factor level of the 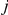 th prognostic factor for the 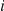 th subject (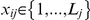). Let 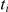 denote the group to which the 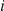 th subject is allocated, 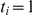 if to the treatment group, and 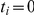 if to the control group. Let 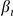 denote the treatment effect, 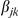 (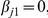 by definition), the effect of the 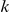
th level of the 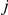 th prognostic factor. We generate the trial response for the 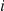 th subject from a normal distribution with unit variance and a mean of 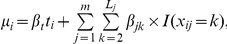 where 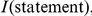 an indicator function, is 1 if the statement is true and 0 if otherwise.

In the simulation, the treatment effects are set at 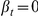 (for examining the type I error rates), 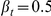 (for powers) and 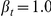 (for powers), respectively. As for the prognostic factors, we examine three scenarios: 1) three binary prognostic factors; 2) six binary prognostic factors; and 3) three polytomous prognostic factors, with number of levels of 5, 4 and 3, respectively. For a binary prognostic factor, the probability of observing a non-reference level is generated from a uniform[0.2, 0.8] distribution. For a polytomous prognostic factor, we assume equal chances of observing any of its levels. The factor levels, 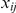
's (for 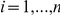 and 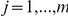), are then generated from the corresponding binomial distributions (for binary prognostic factors) or multinomial distribution (for polytomous prognostic factors), respectively. The effects, 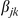
's (for 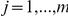 and 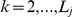), are generated from a uniform[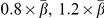] distribution, where 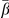 is the average effect of the prognostic factors. In the simulation, 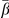 is examined for various values.

We consider two different sample sizes: 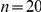 and 40. A total of 10,000 simulations are performed for each scenario. (To estimate a p-value with the absolute relative error median level no larger than 5%, the number of simulations should be no less than 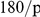 [8]. With p
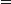
0.05, the number is 3600, justifying our use of 10,000 simulations.)

In each round of the simulation, we perform a multiple linear regression with the dependent variable being the trial response, and the independent variables, the 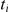 and the 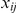
's. (If a prognostic factor has more than two levels, say a total of 5, we enter all its 4 dummy variables into the regression model.) The estimate of the treatment effect and its p-value are recorded. The bias is calculated as the difference between the mean of the estimates and its true value. The variance is calculated as the empirical variance of the estimates across the 10,000 simulations. For comparison, we also calculate the average of the estimated variances from the multiple linear regression. The type I error rate (under the null hypothesis: 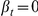) and power (under the alternative hypothesis: 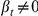) are calculated as the proportion of the simulations with the treatment-effect p-value<0.05.

In addition to the power and the variance described above, predictability of treatment allocation is also an important criterion for evaluating a trial (especially when perfection in masking/concealment is difficult to achieve). If the allocation in a trial can somehow be predicted, the study will be prone to selection bias. In our simulation study, we derive two indices of predictability: Predictability-I: defined as the probability that the next subject is allocated to the group different from the one the previous subject allocated to; and Predictability-II: defined as the probability that the next subject is allocated to the group with fewer subjects already allocated to.

### Simulation Results

**Figure 1:**
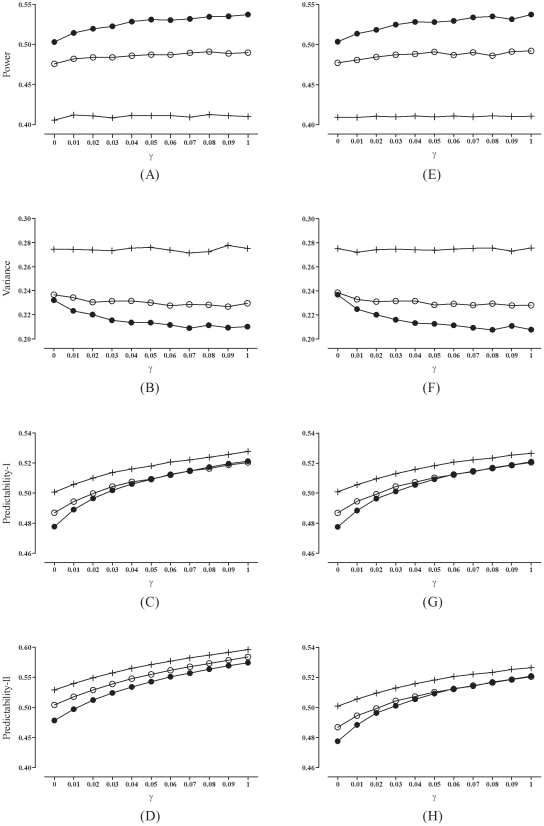
Performances of the two-way minimization using different 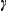 values, under a smaller sample size of 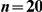 (left panels, A∼D: the average effect of the prognostic factors is smaller, 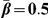; right panels, E∼H: the average effect of the prognostic factors is larger, 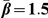; solid circle: with three binary prognostic factors; hollow circle: with six binary prognostic factors; cross: with three polytomous prognostic factors.

**Figure 2:**
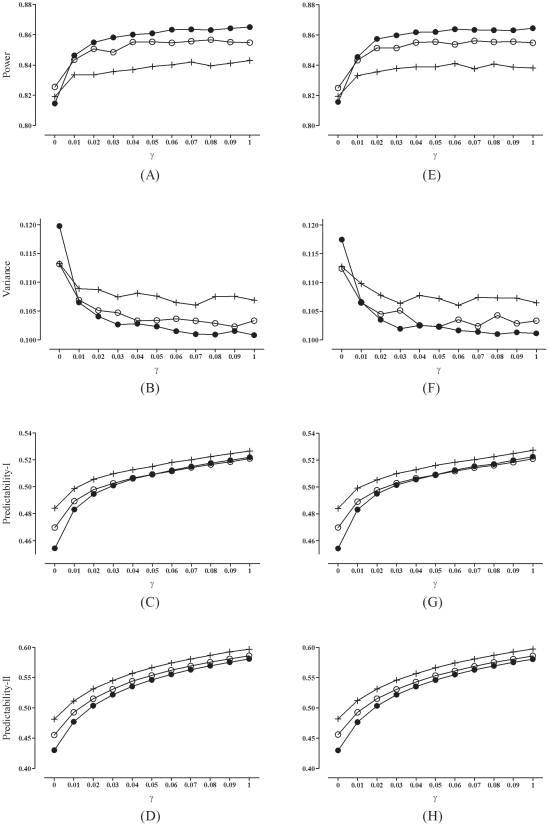
Performances of the two-way minimization using different 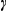 values, under a larger sample size of 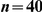 (left panels, A∼D: the average effect of the prognostic factors is smaller, 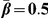; right panels, E∼H: the average effect of the prognostic factors is larger, 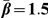; solid circle: with three binary prognostic factors; hollow circle: with six binary prognostic factors; cross: with three polytomous prognostic factors.

Figure 1 shows the performances (when 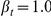) of the two-way minimization using different 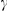 values (0 to 0.1, by 0.01), under a smaller sample size of 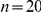. Figure 2 shows the corresponding performances under a larger sample size of 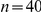. From both figures, we see that to have better statistical performances (higher power and smaller variance), one should choose a 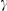 value that is larger. On the other hand to make the allocation less predictable, one should choose a 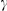 value that is smaller. Taken together, we settle on 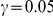 as a satisfactory compromise.

Table 1 shows the biases and variances for the two-way minimization with 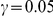. We see that the two-way minimization produces approximately unbiased estimates of the treatment effects. We also see that the averages of the estimated variances closely match with the corresponding empirical variances of the estimates, indicating that the standard estimates of variances in a multiple linear regression (with 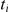 and dummy codes of 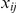
's as regressors) are accurate, even for a complex allocation scheme such as the two-way minimization. For hypothesis testing of the treatment effect (at a significance level of 0.05), Table 2 shows that the two-way minimization can maintain quite accurate type I error rates, and that its power increases as the treatment effect increases.

**Table 1:** Biases and variances for the two-way minimization with 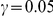.

Sample Size	Number and Type of Prognostic Factors	Treatment Effect		
		0.0	0.5	1.0
Bias				
20	Three binary prognostic factors	0.0031	0.0013	0.0073
	Six binary prognostic factors	−0.0042	0.0007	−0.0012
	Three polytomous prognostic factors	−0.0061	0.0007	0.0016
40	Three binary prognostic factors	−0.0095	−0.0021	0.0023
	Six binary prognostic factors	0.0052	0.0032	−0.0012
	Three polytomous prognostic factors	−0.0039	0.0032	−0.0028
Variance of estimates/Average of estimated variances				
20	Three binary prognostic factors	0.2117/0.2123	0.2136/0.2124	0.2112/0.2128
	Six binary prognostic factors	0.2306/0.2287	0.2253/0.2298	0.2289/0.2270
	Three polytomous prognostic factors	0.2699/0.2741	0.2758/0.2711	0.2773/0.2720
40	Three binary prognostic factors	0.1009/0.1016	0.1029/0.1017	0.1042/0.1018
	Six binary prognostic factors	0.1024/0.1034	0.1011/0.1031	0.1036/0.1035
	Three polytomous prognostic factors	0.1109/0.1080	0.1081/0.1073	0.1059/0.1074

**Table 2:** Type I error rates and powers at a significance level of 0.05 for the two-way minimization with 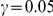.

Sample Size	Number and Type of Prognostic Factors	Type I Error Rate	Power	
			Treatment Effect = 0.5	Treatment Effect = 1.0
20	Three binary prognostic factors	0.0491	0.1738	0.5338
	Six binary prognostic factors	0.0511	0.1569	0.4946
	Three polytomous prognostic factors	0.0511	0.1457	0.4154
40	Three binary prognostic factors	0.0495	0.3339	0.8639
	Six binary prognostic factors	0.0483	0.3268	0.8537
	Three polytomous prognostic factors	0.0507	0.3192	0.8421

**Figure 3:**
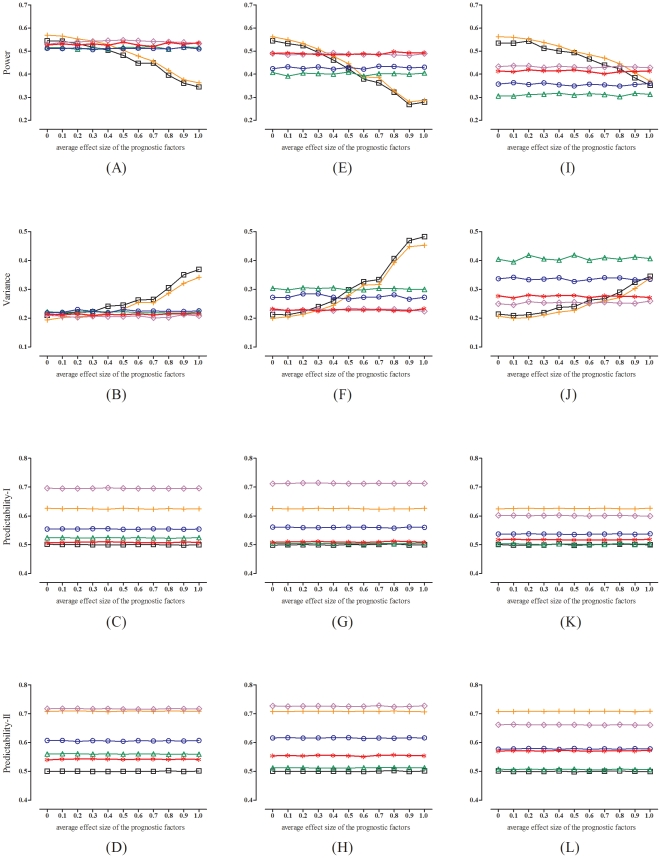
Performances of the two-way minimization with 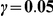 (red star), as compared to those of the simple randomization (black square), the block randomization with block size = 4 (orange cross), the stratified randomization with block size = 4 (green triangle), the deterministic minimization (purple rhombus), and the biased coin minimization with coin probability = 0.7 (blue circle), under a smaller sample size of 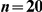 (left panels, A∼D: with three binary prognostic factors; middle panels, E∼H: with six binary prognostic factors; right panels, I∼L: with three polytomous prognostic factors). The treatment effect is set at 1.0.

**Figure 4:**
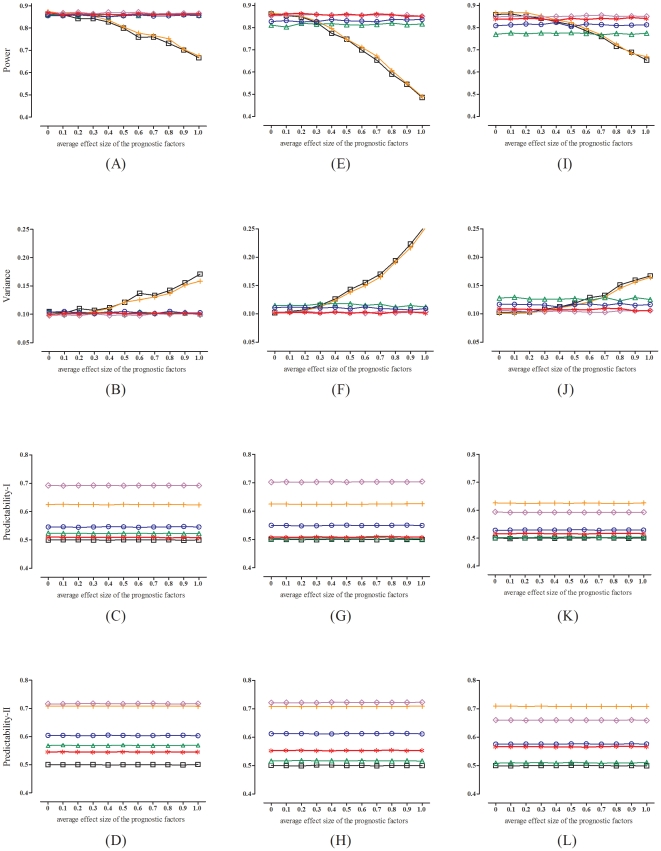
Performances of the two-way minimization with 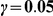 (red star), as compared to those of the simple randomization (black square), the block randomization with block size = 4 (orange cross), the stratified randomization with block size = 4 (green triangle), the deterministic minimization (purple rhombus), and the biased coin minimization with coin probability = 0.7 (blue circle), under a larger sample size of 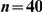 (left panels, A∼D: with three binary prognostic factors; middle panels, E∼H: with six binary prognostic factors; right panels, I∼L: with three polytomous prognostic factors). The treatment effect is set at 1.0.

Figure 3 compares the performances (when 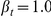) of the two-way minimization (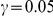) with five other allocation methods: the simple randomization, the block randomization (block size
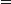
4), the stratified randomization (block size
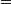
4), the deterministic minimization, and the biased coin minimization (coin probability
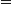
0.7), under a smaller sample size of 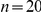. Figure 4 presents the corresponding results under a larger sample size of 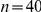. We see that as the average effect of the prognostic factors increases, the performances (in terms of power and variance) of the simple randomization and the block randomization run down quickly, whereas the performances of the four methods that balance prognostic factors (the stratified randomization, the deterministic minimization, the biased coin minimization, and the two-way minimization) remain fairly stable. However, when there are more prognostic factors (panels E and F) or more factor levels (panels I and J) to be balanced (as compared to the situation of three binary prognostic factors, panels A and B), the performances of the stratified randomization and the biased coin minimization deteriorate. By contrast, the deterministic minimization and the two-way minimization suffer very little performance loss, if they are charged with balancing more prognostic factors or more factor levels.

As for the allocation predictability (panels, C, D, G, H, K and L, in Figures 3 and 4), we see that the deterministic minimization and the block randomization are rather predictable. With these two methods, an artful patient can have a 70∶30 chance of getting what he/she desires. The biased coin minimization shows some improvement, though it is still not good enough (predictability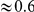). To have a satisfactory control of the selection bias, one needs to turn to the stratified randomization or the two-way minimization (predictability
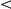
∼0.55), or to eliminate it completely, to the gold-standard simple randomization (predictability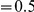).

## Discussion

In this study, we focused on trials with small sample sizes. We showed that the proposed two-way minimization has the correct type I error rates. It also produces point estimates that are unbiased and variance estimates that are accurate. We compared the performances of the new method with several existing methods. Four methods can maintain stable performances as the effects of prognostic factors increase, namely: 1) the stratified randomization; 2) the biased coin minimization; 3) the deterministic minimization; and 4) the proposed two-way minimization. However, the first three methods have drawbacks: the stratified randomization and the biased coin minimization perform less than ideally when they are charged with balancing more prognostic factors/levels; the deterministic minimization is rather easy to predict and is therefore prone to selection bias. By comparison, the proposed two-way minimization is a better method for balancing prognostic factors in small trials.

For a large trial, it is generally held that even a simple randomization suffices. But there is no reason why one cannot force balance a large trial using the two-way minimization. In fact in doing so, he/she will be rewarded with even higher statistical performances as compared to leaving everything to chance. For example in a trial with 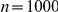 and six binary prognostic factors, the powers are 0.6612 (two-way minimization) and 0.6113 (simple randomization), the variances are 0.0039 (two-way minimization) and 0.0045 (simple randomization), when the treatment effect is 0.15 and the effect of the prognostic factors is 0.3.

The two-way minimization may appear to be a fancy allocation procedure that is unduly complex. Yet, the entire algorithm of it can actually be incorporated into a simple spreadsheet program (available from the authors). Then, all that a trial researcher has to do is to simply feed in the prognostic-factor information for the subjects consecutively recruited in the trial. The allocation for them shall be produced one by one from the program fully automatically. The two-way minimization also calls for simple analysis despite its complex allocation scheme—a regression adjustment for the force-balanced prognostic factors is all that is needed. Further studies are warranted to extend the two-way minimization to deal with unbalanced designs where the treatment and the control groups are not to be of equal sample size due to ethical or logistical considerations. More work is also needed to study the performances of two-way minimization for other types of trial response, such as non-normal, binary, Poisson, and time-to-event data, etc, and whether the optimal value for the tuning parameter of 0.05 that was identified remains optimal for these other response types.

Recently, Perry et al. [9] also proposed an improved minimization method, the ‘studywise minimization’. The method exhaustedly searches among all possible allocations in a trial for one that leads to minimum imbalance. It also has virtue of being nearly unpredictable. However, the allocation of subjects (and also the administering of the treatment) in that method has to be deferred until all subjects intended for study has been recruited. This essentially excludes its applicability in trials with extended recruitment period and for treatments which must be immediately given once subjects are recruited.

In conclusion, the proposed two-way minimization has desirable statistical properties and is resistant to selection bias. The allocation can be done in real time and the subsequent data analysis is straightforward. The two-way minimization is recommended to balance prognostic factors in small trials.
